# Chemolithoautotrophic bacteria flourish at dark water–ice interfaces of an emerged Arctic cold seep

**DOI:** 10.1093/ismejo/wrae170

**Published:** 2024-09-12

**Authors:** Lisa-Marie Delpech, Alexander T Tveit, Andrew J Hodson, Kevin P Hand, Dimitri Kalenitchenko

**Affiliations:** LIENSs Littoral Environnement et Sociétés, UMRi 7266 CNRS–La Rochelle Université, La Rochelle, 17000, France; Department of Geosciences, UiT The Arctic University of Norway, Tromsø, 9010, Norway; Department of Biology, École Normale Supérieure de Lyon, Lyon, 69007, France; Department of Arctic and Marine Biology, UiT The Arctic University of Norway, Tromsø, 9019, Norway; Department of Arctic Geology, UNIS The University Center in Svalbard, Longyearbyen, 9170, Svalbard, Norway; Department of Civil Engineering and Environmental Science, Western Norway University of Applied Sciences, Sogndal, 6856, Norway; Jet Propulsion Laboratory, California Institute of Technology, Pasadena, CA, 91109, United States; LIENSs Littoral Environnement et Sociétés, UMRi 7266 CNRS–La Rochelle Université, La Rochelle, 17000, France; Department of Geosciences, UiT The Arctic University of Norway, Tromsø, 9010, Norway

**Keywords:** chemoautotrophs, methane, sulfur, cold seep, Arctic, terrestrial ice

## Abstract

Below their ice shells, icy moons may offer a source of chemical energy that could support microbial life in the absence of light. In the Arctic, past and present glacial retreat leads to isostatic uplift of sediments through which cold and methane-saturated groundwater travels. This fluid reaches the surface and freezes as hill-shaped icings during winter, producing dark ice–water interfaces above water ponds containing chemical energy sources. In one such system characterized by elevated methane concentrations — the Lagoon Pingo in Adventdalen, Svalbard, Norway (~10 mg/L CH_4_, <0.3 mg/L O_2_, −0.25°C, pH 7.9), we studied amplicons of the bacterial and archaeal (microbial) 16S rRNA gene and transcripts in the water pond and overlaying ice. We found that active chemolithoautotrophic sulfur-oxidizing microorganisms (*Sulfurimonas*, *Thiomicrorhabdus*) dominate a niche at the bottom of the ice that is in contact with the anoxic water reservoir. There, the growing ice offers surfaces that interface with water and hosts favorable physico-chemical conditions for sulfide oxidation. The detection of anaerobic methanotrophs further suggests that throughout the winter, a steady-state dark and cold methane sink occurs under the ice in two steps: first, methane is oxidized to carbon dioxide and sulfates are concomitantly reduced to sulfides by the activity of anaerobic methanotrophs (ANME) ANME-1a and sulfate-reducing bacteria (SRB) SEEP-SRB1 consortia; and second, energy from sulfides is used by sulfur-oxidizing microorganisms to fix carbon dioxide into organic carbon. Our results underscore that ice-covered and dark ecosystems are hitherto overlooked oases of microbial life and emphasize the need to study microbial communities in icy habitats.

## Introduction

Ice–water interfaces are targets in the search for life beneath the ice shells of icy moons because these interfaces may contain oxidants required for redox reactions, potentially available through ice irradiation [1]. On Earth, ecosystems that mirror the features of liquid oceans of icy moons such as Europa and Enceladus — characterized by the absence of light and presence of a chemical energy source in the form of methane and hydrogen sulfide [1] — are often encountered in the deep sea [2] or in terrestrial sulphidic environments such as cold springs [3, 4], cave ecosystems [5], or even stratified temperate lakes [6]. However, environments that also harbor ice–water interfaces are scarce [7]. These environments can exist under the ice of polar lakes characterized by a source of biogenic methane [8, 9]. This methane, together with carbon dioxide, is the final product of organic matter degradation by methanogenic archaea in sediments [10–12] and can also be recharged from the active layer of the permafrost [13]. The presence of methane supports the development of methanotrophs in sediments [14] and in the water column [15] as members of microbial consortia that often produce reduced sulfur-containing molecules as a by-product of anaerobic methane oxidation [14].

At Moskuslaguna on the Svalbard coast (Adventdalen, 78°N, Norway), geological structures specific to deglaciated landscapes harbor relevant features for studying life in the absence of light. There, groundwater seepage creates hydraulic pressure within the permafrost, leading to the formation of hill-shaped structures called open-system pingos [16]. In this environment, seasonal ice overlies an artesian source of reduced fluid originating below the permafrost. The reduced fluid is loaded with greenhouse gases from the anaerobic microbial degradation of permafrost organic matter or from the dissociation of gas hydrates [17]. At the Lagoon Pingo in Adventdalen, Svalbard, Norway (78°14′22.5 N; 015°45′16.0 E), an ice blister forms every winter above the reduced fluid seepage. This 1-m-thick ice layer encapsulates a pressurized and light-deprived water reservoir (<10-m-wide, 1-m-deep; Supplementary Fig. 1) that is saturated with methane (up to 0.9 mM) [16]. Unlike polar lakes for which the most common sources of methane and hydrogen sulfide are autochthonous production by methanogenesis and sulfate reduction (e.g. up to 1.5 μM under the ice [18]), at Lagoon Pingo the allochthonous supply of reduced fluid generates methane concentrations 1000 times higher. Sulfate concentrations in excess of those recorded in surface waters of the regions and occasional odors of H_2_S indicate a significant role of the microbial sulfur cycle [16], making this an ideal system to study chemolithoautotrophic psychrophilic organisms and the possibility of life in icy, light-deprived habitats.

The cryosphere is far from biologically inactive. Brine channels in sea ice harbor a microbial oasis where, despite low temperatures, photoautotrophic microorganisms bloom at the ice–water interface when the light returns to the Earth’s poles, triggering a solar energy-based food web [19, 20]. Microbial life is found where liquid water is available, such as in cryoconite holes, glacier ice surfaces, or subglacial environments where high carbon content, bedrock weathering, and oxygen from glacial meltwater provide energy for microbial metabolism, highlighting the importance of interfaces [21]. Subglacial environments are one example of a light-deprived icy environment; however, they are difficult to access. In other terrestrial icy environments devoid of light, such as ice-covered lakes, previous studies have highlighted the potential role of ice in hosting life based on methane oxidation, yet the microbiome of the ice associated with these systems remains to be investigated [18, 22, 23]. In this study, we explored whether the ice shell of the Lagoon Pingo could host microbial life supported by a chemical source of energy.

## Materials and methods

The sampling design and methods used in this study are summarized in the main text and further described in the Supplementary Information.

## Results and discussion

In March 2021, we collected three ice cores from the ice blister above the fluid seepage (Supplementary Fig. 1) and investigated microbial abundance and microbial community composition using quantitative polymerase chain reaction and 16S rRNA gene and transcript sequencing. The water in the reservoir was anoxic to microoxic, with an O_2_ concentration below 0.3 mg/L and a strong reductive potential (−371.4 mV). The high electrical conductivity (6.3 mS/cm) relative to natural surface waters of the region indicated that highly concentrated water was feeding the pond [16]. A pH of 7.9 indicated that the H_2_S, whose presence was assumed from the odor escaping the pond when drilling (Supplementary Fig. 2), was mostly in its ionic bisulfide form (HS^−^).

**Figure 1:**
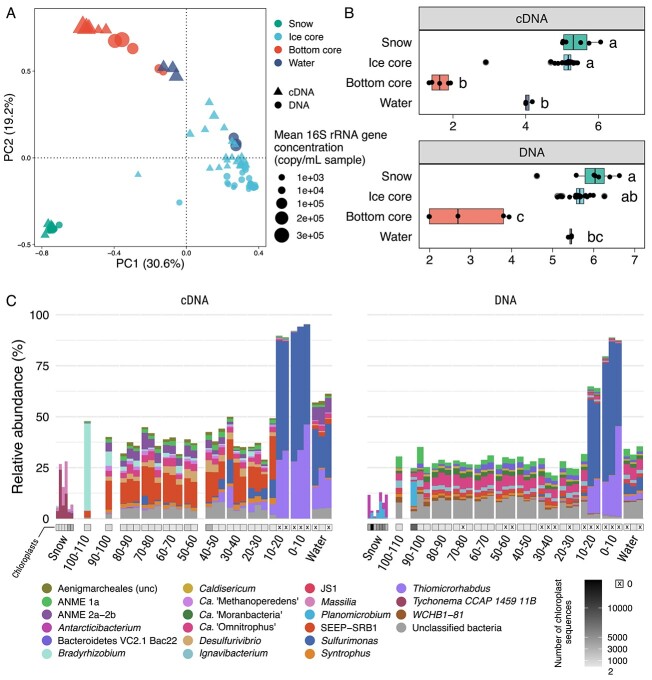
(**A**) Principal component analysis showing bacterial and archaeal beta diversity in the Lagoon Pingo ice-covered system. Colors show the sample’s environment (snow, water, ice), and the bottom core was defined based on the dominance of *Sulfurimonas*. The size of the points show the bacterial 16S rRNA gene concentration in copy number/mL of sample, as determined by quantitative polymerase chain reaction. (**B**) Shannon index for the four environments presented in (A). Letters indicate groups of significance (False Discovery Rate-corrected *P* value <.05) representing Dunn’s test output after Kruskal-Wallis significance tests. Observed ASV numbers are available in Supplementary Fig. 4. (**C**) Genus affiliation of the 20 most abundant ASVs in the cDNA (left) and DNA dataset (right). The *x* axis shows the height of the ice core (in cm) relative to the water–ice interface (0 cm). Shades of grey below the bars indicate the number of chloroplast reads identified in the sample; a cross on white background indicates the absence of chloroplast read. ASV, amplicon sequence variant.

The three ice cores showed similar exponential increases in microbial 16S rRNA gene abundances with depth. The bottom 10 cm of the core exhibited a mean three-fold increase in 16S rRNA gene concentration (2.5 × 10^5^ ± 9.9 × 10^4^ copies/mL, *n* = 3) compared to the water reservoir (9.3 × 10^4^ ± 2.9 × 10^4^ copies/mL, *n* = 3), and a 100-fold increase compared to the top first 80 cm of the core (2.4 × 10^3^ ± 2.4 × 10^3^ copies/mL, *n* = 3) (Fig. 1A and Supplementary Fig. 3). Therefore, we concluded that a microbiologically active layer exists within 0 to 20 cm of ice from the interface with the water reservoir, although the actual activity of these bacteria within the ice remains uncertain [24]. Concurrently, Shannon’s diversity index dropped in the bottom core (defined in the Supplementary Information) compared to the core top (*P* value <.001) and underlying water reservoir (*P* value not significant) (Fig. 1B). This indicates the presence of a singular specialized ecological niche in the bottom ice.

The exponential increase of microbial abundance with depth in our samples resembles the increase in diatom abundances found in sea ice brine channels at the ocean–ice interface [19]. However, at the time of sampling, light had returned for only two weeks. The absence of chloroplast reads (Fig. 1C) and the presence of very low cyanobacteria relative abundances (< 0.025% DNA and cDNA in the bottom core) (Supplementary Fig. 5) attests to the lack of photosynthetic activity at the ice–reservoir interface. The bottom core community was, in fact, dominated by three amplicon sequence variants (ASVs) associated with the sulfur-oxidizing bacteria (SOB) genera *Sulfurimonas* (ASV_1, ASV_9) and *Thiomicrorhabdus* (ASV_2) (Fig. 1C), which accounted for 53% to 85% of the community (DNA) and 86% to 95% of the active community (cDNA). In the water, the ratio between the cDNA and DNA relative abundances of ASVs affiliated with SOBs was five times that in the bottom ice, indicating higher SOB activity in the reservoir (Supplementary Fig. 6). The reduced relative abundance in the DNA dataset suggests that SOBs might be limited in space in the water reservoir, whereas the bottom core likely provides surfaces in contact with water, suiting their niche.

**Figure 2:**
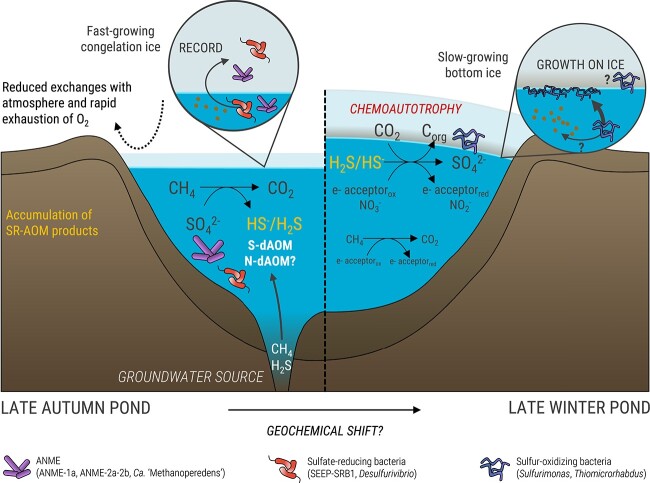
Conceptual figure depicting the hypothetical functioning of the system during the ice-covered period. Left panel: Microbiological processes in the pingo water reservoir and ice cap just after the onset of the ice-cover formation, as hypothesized using the communities registered in the ice core. Right panel: Accumulated reduced sulfur species provide energy for SOB-mediated chemolithoautotrophy, which find a niche at the water–ice interface when ice has a slow growth at the end of the ice-covered period. SR, Sulfate reduction; AOM, anaerobic oxidation of methane; N-dAOM, nitrate-dependent AOM; S-dAOM, sulfate-dependent AOM.

Phylogenetic placement of ASVs affiliated with *Sulfurimonas* showed multiple disagreeing placements. Likelihood weight ratios were between 0.02 and 0.33 (ASV_1, *n* = 4 placements), and 0.05 and 0.60 (ASV_9, *n* = 4). The branch length indicated distant similarities with known species of the genera *Sulfurimonas* and *Sulfuricurvum*, suggesting that these two ASVs are related to SOBs but are likely from unknown species or genera (Supplementary Fig. 7 and Supplementary Table 1). ASV_2, representative of the *Thiomicrorhabdus* population inhabiting the bottom core, was closely related to *Thiomicrorhabdus aquaedulcis* [25] (Supplementary Fig. 7) (98.93% nucleotidic similarity, likelihood weight ratio = 0.99, *n* = 1). *Sulfurimonas* and *Thiomicrorhabdus* genera mostly consist of aerobic chemolithoautotrophs, which generally use O_2_ as the electron acceptor [26, 27]. Despite limited oxygen availability in the water, these species might benefit from punctual microoxic conditions through ice fissures, as efficient microaerophilic growth is reported for some *Sulfurimonas* spp. [28]. Alternative electron acceptors for *Sulfurimonas* spp. and related species include nitrate, nitrite [27, 29, 30], and manganese oxides [31]. One *Thiomicrorhabdus* sp. was reported to be capable of growing in anoxic conditions using nitrite as an electron acceptor [32]. In the Lagoon Pingo, nitrate concentrations are below detection limits in the reservoir [16]. This depletion could be explained by rapid use of nitrate by these SOBs and/or by denitrification involving *Ca*. “Methanoperedens” [33], which was consistently observed in the ice and water reservoir (Fig. 1C and Fig. 2).

Several factors could explain the repartition of sulfur oxidizers in the bottom core (Fig. 2): (1) electron acceptors for sulfur oxidation might be more available, potentially through cracks in the ice (O_2_) or background photosynthesis enabling efficient use of other electron acceptors under microoxic conditions as reported for many *Sulfurimonas* spp. [28]. (2) A shift in the reservoir geochemistry or redox balance — e.g. accumulation of reduced sulfur species, allowing SOBs to thrive at the end of the ice-covered period. However, the import or accumulation of electron acceptors and donors would benefit the reservoir’s communities as well and cannot explain the observed higher abundance of bacteria in the bottom ice versus water reservoir. (3) The ice structure might provide more surfaces for bacterial attachment. Young bottom ice forms much more slowly later in winter than at the beginning of the freezing process [34], leaving enough time for bacteria to settle on these surfaces [23]. This finding is consistent with the reported ability of *Sulfurovum*/*Sulfurimonas*-related and *Thiomicrorhabdus* spp. to attach to surfaces [35, 36] and the overall tendency of SOBs to form biofilms [37–39]. These results will need confirmation by in-depth studies confronting the geochemistry and microbiology of the ice and the reservoir throughout the winter period.

Reduced sulfur compounds (HS^−^) feeding sulfur oxidation could be sourced from the fluid or produced in the reservoir by consortia of ANMEs and SRBs. The concomitant presence of ANMEs and SRBs in the first 80 cm of the ice and in the water suggests that microbial anaerobic oxidation of methane (AOM) and sulfate reduction occurred throughout winter in the reservoir, with the water acting as a liquid incubator for ANMEs and SRBs (Fig. 2) that turn the system into a methane sink during the ice-covered period. Relative abundances of ANME-1a and SEEP-SRB1 were strongly correlated (Supplementary Fig. 8), suggesting that sulfate-dependent AOM (S-dAOM) was occurring [40]. The lower relative abundance of SRBs and higher abundance of ANME-2a-2b in the active community of the reservoir compared to the ice (Fig. 1C) suggest that S-dAOM likely occurred until a shift in the system opened a niche for ANMEs that use other electron acceptors. The discrepancy between DNA and cDNA relative abundances of SEEP-SRB1 (Fig. 1C**,**Supplementary Fig. 6) further suggests that the ice core recorded a seasonal shift in the reservoir, initially favoring sulfate reduction until SOBs dominated the community and contributed to the carbon sink through chemolithoautotrophy. More generally, the pool of reduced sulfur compounds might enhance carbon sequestration, as sulfurization of dissolved and particulate organic carbon generates refractory organic matter in oxygen-deprived environments [41].

This study demonstrated the significant role of the ice cap above a chemical energy-rich groundwater seepage in hosting active chemotrophic microorganisms that consume greenhouse gases. We found active anaerobic methanotrophs in the water reservoir, indicating a dynamic winter system where at least part of the greenhouse gases brought by the source is oxidized under the ice. An active community of SOBs thrives on the ice at the water interface. This finding strongly suggests that active microbial habitats at ice–water interfaces in dark environments have been overlooked and may provide valuable insights to the field of microbiology.

## Supplementary Material

METHANICE1_BriefComm_ISME_SI_FinalRevision_wrae170

## Data Availability

The sequences reported in this paper have been deposited in the NCBI SRA database under the accession numbers SRR24977479–SRR24977560. Reproducible code for the figures and results presented in this study and in the Supplementary Information is available at https://github.com/lmdelpech/MethanIce-BriefReport-LP-ICE-2021.
