# Supplementary material for: Chemolithoautotrophic bacteria flourish at dark water–ice interfaces of an emerged Arctic cold seep
Source: ISME J. 2024 Sep 12;18(1):wrae170. doi: 10.1093/ismejo/wrae170 (PMC11637994; doi:10.1093/ismejo/wrae170)
Supplement: METHANICE1_BriefComm_ISME_SI_FinalRevision_wrae170 [file methanice1_briefcomm_isme_si_finalrevision_wrae170.pdf]

Supplementary Information for

Chemolithoautotrophic bacteria flourish at dark water-ice interfaces of an emerged Arctic cold seep

Short title: Frozen cold seep hosts chemoautotrophs

Authors: Lisa-Marie Delpech[1,2,3*], Alexander T. Tveit[4], Andrew J. Hodson[5,6], Kevin P. Hand[7], Dimitri Kalenitchenko[1,2]

1. Littoral Environnement et Sociétés (LIENSs), UMRi 7266 CNRS - La Rochelle Université, La Rochelle 17000, France
2. Department of Geosciences, UiT The Arctic University of Norway, Tromsø, Norway
3. Department of Biology, École Normale Supérieure de Lyon, Lyon 69007, France
4. Department of Arctic and Marine Biology, UiT The Arctic University of Norway, Tromsø, Norway
5. Department of Arctic Geology, UNIS The University Center in Svalbard, Longyearbyen, Svalbard, Norway
6. Department of Civil Engineering and Environmental Science, Western Norway University of Applied Sciences, Sogndal, Norway
7. Jet Propulsion Laboratory, California Institute of Technology, CA 91109, Pasadena, USA

*Corresponding author: Lisa-Marie Delpech, **email**: lisa-marie.delpech@univ-lr.fr

This PDF file includes:

Supporting text
Supporting figures: Supplementary Figure 1 to 8
Supporting tables: Supplementary Table 1
SI References

Supporting Information Text

Study site and field sampling

The Lagoon Pingo (N78°14'22 E015°45'16) is an open system pingo located in the outermost part of Adventdalen, Svalbard. The pingo is located at the shore, on the Northern side of Adventdalen river, separated (600 m) from tidal influence a lagoon (Moskuslagoon). The system is hypothesized to have originated as a marine pockmark [1], turned terrestrial due to isostatic uplift caused by glacial retreat.

The system consists of several elevated mounds with craters tracing the inner edge of the Moskuslagoon. The groundwater spring, which is defined as the primary fluid source in this study, emerges from the northernmost crater named Lagoon Pingo East (LPE). The average groundwater discharge in LPE is 0.1-1 $L.s^{-1}$ of brackish, oxygen-depleted water [1]. LPE is seasonally ice-covered with an ice blister that reaches ca. 1 m thickness in winter, itself covered by snow. This ice blister creates hydrostatic pressure, *i.e.* the water outflows when the ice is cracked open. Freezing starts in late October, and the ice collapses in early June.

Sampling was conducted on March 1st, 2021. At that time, the pingo was covered with an ice lid of 1 m thickness and ca. 25 cm of snow. Seven snow samples were collected, after what the snow was ablated for ice core sampling. Three 14 cm-wide ice cores were collected at 2 m intervals, using a Mark V coring system (Kovacs) and were surface-decontaminated using 5% sodium hypochloride to prevent contamination from the corer or the surrounding environment. Cores were sliced on site into 10 cm sections using an ethanol-washed handsaw. Core 1 and core 3 were 100 cm thick, core 2 was 110 cm thick. The 10 cm sections were vacuum packed on site using a food sealer. Snow and ice samples were melted back at UNIS facilities at room temperature and filtration was carried out as soon as the ice had completely melted. Each melted snow and ice volume was filtered on a 0.22 µm Sterivex unit (MF-Millipore Membrane, MA, USA), using a peristaltic pump. Three water samples were collected from the upper layer of the water reservoir, directly underlying the ice blister. Sterivex filters were stored in closed sterile tubes at -80°C until further processing and shipped back to UiT in dry ice (-74°C).

The dissolved oxygen concentration (DO), oxidation-reduction potential (ORP), conductivity and pH of the water were determined immediately on site from a borehole whilst samples were being taken. The measurements used Hach Lange HQ40d multiparameter meters with a new, factory calibrated fluorescence detector (for O_2, detection limit 0.1 mg/L) and gel electrode (for ORP and pH).

DNA extraction, library preparation and sequencing

Sterivex™ filters were cut open under a sterile, UV-cleaned bench in order to improve extraction yields [2]. The filter was cut into pieces and half of it was put into a Lysing Matrix E 2 mL tube (MP Biomedicals, California, USA). Total NA extraction was then conducted applying a phenol/chloroform isolation method (adapted after [3]). Lysing Matrix E tubes containing the samples were briefly thawed on ice and 500 µL of Phenol:Chloroform:Isoamylalcohol (25:24:1, pH 8) solution and TNS extraction buffer (10% SDS, 500 mM TRIZMA, 100 mM NaCl, 0.1% DEPC) were added. The tubes were bead-beaten for 30 sec at 5.0 $m.s^{-1}$ using an MP Biomedicals FastPrep-24 (California, USA), cooled with dry ice in the adaptor head. Samples were centrifuged at 13 000 g and 4°C for 10 minutes. The supernatant was transferred to a clean tube and extracted with one volume of Chloroform:Isoamylalcohol (24:1). Samples were inverted several times, centrifuged at 13 000 g and 4°C for 10 min, the supernatant collected to a clean tube and precipitated with two volumes of PEG precipitation solution (30% polyethylene glycol MW 7000-9000, 1.6 M NaCl, 0.1% DEPC) and 0.003 volume of glycogen (Invitrogen by ThermoFischer, 5 mg/mL, final concentration 0.015 mg/mL). Samples were inverted several times, left for precipitation on ice for 1 h and centrifuged at 13 000 g and 4°C for 1 h. The TNA pellet was washed twice with 1 mL of ice-cold 70% ethanol, dried for 3 min at 55°C and eluted with 50 µL of DEPC-treated water and 0.5 µL RNase inhibitor (Invitrogen by ThermoFischer).

TNA extracts were sent to IMGM Laboratories GmbH (Planegg, Germany) for amplification and sequencing of a ca. 411 bp fragment in the V4-V5 region of the 16S rRNA gene, using primers 515F-Y (5'-GTGYCAGCMGCCGCGGTAA) and 926R (5'-CCGYCAATTYMTTTRAGTTT) [4].

cDNA libraries of the same region of the 16S rRNA gene were obtained from an additional RT-PCR step. Sequencing was performed on a MiSeq sequencing system (Illumina) with 2x300 bp paired-end reads chemistry.

Sequence bioinformatic processing

DADA2 [5] implemented in Qiime2 (v2021.8.0) was used to filter, merge, denoise and remove chimeric sequences of raw 16S amplicon reads with truncation length of 270 (forward) and 210 (reverse) bp. The average percentage of input reads retained per sample after this pipeline was 54.6% for cDNA and 67.0% for DNA. Taxonomy of the resulting inferred ASVs was assigned using Mothur (v1.43.0) [6] with the version 138 of the SILVA nonredundant SSU database [7]. ASVs assigned to Eukaryota, mitochondria, chloroplasts or whose taxonomy was not assigned at the domain level were removed prior to analysis. This step further removed 0.89% of cDNA and 2,26% of DNA retained ASVs. Sequences present in the target-specific no-target control sample conducted during the PCR amplification process were identified as contaminant and substracted from the ASV table. After those steps, 19 338 (14 534) ASVs were left in 41 DNA (cDNA) samples, with a range of 39 255 (533) to 197 705 (178 379) sequences per sample. DNA and cDNA datasets were rarefied to a depth of 39 255 (24 241) sequences, leaving 18 637 (12 231) ASVs in 41 (37) samples respectively. A dataset rarefied to a depth of 24 241 sequences per sample was generated to compare DNA and cDNA datasets in diversity analyses.

Quantitative Polymerase Chain Reaction (qPCR) analysis

The abundance of 16S rRNA gene from Bacteria was estimated using quantitative PCR (qPCR) with the primers BACT1369F (CGGTGAATACGTTCYCGG) and PROK1492R (GGWTACCTTGTTACGACTT) [8]. The quantification was performed in triplicates, using different DNA concentrations to compensate for PCR inhibition. Amplification reactions were carried in a CFX96™ Real-Time system (Bio-Rad Laboratories, Hercules, CA, USA), in a final volume of 20 µL using Sso Fast™ EvaGreen® Supermix (Bio-Rad Laboratories, Hercules, CA, USA). qPCR conditions were as follow: 40 cycles of denaturation at 94°C for 25 sec, annealing at 56°C for 20 sec, elongation at 72°C for 45 sec, and fluorescence was measured at 82°C for 10 sec. Standard curves were built from dilution series of genomic DNA from *Methylobacter tundripaludum* SV96^{T}, with concentrations ranging from 10^2 to 10^6 copy.µL^{-1} and quantified in triplicates. R^2 were between 0.997 and 0.999, and efficiencies between 99.5% and 110.4%. qPCR results were expressed as 16S rRNA gene copy per milliliter of water or melted ice.

Statistical analyses

Ice samples were examined based on their depth (bottom core *versus* rest of the core) building on previous knowledge [9] and on preliminary exploration of the taxonomic composition of the ice core microbial communities. Bottom core samples were defined as ice core samples where *Sulfurimonas* became the dominant genus in relative abundance. This corresponded to the last 20 cm of the cores, except for Core 1, in which *Sulfurimonas* only dominated the last 10 cm section.

All analyses were performed in R (v4.2.2) [10]. Alpha diversity indices were estimated on rarefied datasets, using the estimate_Richness function in phyloseq (v.1.38.0) [11]. Mean differences of alpha diversity index estimates between groups were tested using Kruskal-Wallis test and pairwise differences were tested using the post hoc Dunn's test on KW test output (false discovery rate corrected *P* values), accounting for non-gaussian and non-homoscedastic data. Observed number of ASVs are available as **Supplementary Figure 4**.

Ordinations were performed using the vegan package in R (v.2.5-7) [12]. Principal Component Analysis (PCA) was computed on Hellinger-transformed rarefied matrices [13]. Multivariate homogeneity of group dispersions was tested using betadisper. To further test for differences in community structure (beta diversity), Permutational Multivariate Analysis of Variance (PERMANOVA) [14] was performed using the adonis function in vegan and the Bray-Curtis distance. Pairwise differences were tested using the method implemented in the pairwiseAdonis package (v.0.4) [15]. 64% of the variation within the DNA dataset was attributed to the environments (snow, ice core, bottom core, water) (PERMANOVA R^2 = 0.64, P value < 0.001, all pairwise comparisons significant). 66% of variation was explained by the same groups in the cDNA dataset (PERMANOVA R^2 = 0.66, P value < 0.001, all pairwise comparisons significant).

Correlations between ANME-1a or ANME-2a-2b and SEEP-SRB1 were computed using Spearman rank test, only considering ice core and water samples for ecological relevance. Spearman's correlations between ANME-1a and SEEP-SRB1 were significant (ρ = 0.37, *P* value < 0.05 for DNA and ρ = 0.65, *P* value <0 .0005 for cDNA), correlations were not significant for ANME-2a-2b and SEEP-SRB1.
All plots were made using ggplot2 in R (v.3.3.6) [16].

Taxonomic composition and phylogenetic placement of ASVs
Taxonomic composition is reported as relative abundances (relative to the sample total sequence abundance). For community overview, the most abundant ASVs (*n* = 20) are represented with their higher taxonomic level affiliation. Sequences reported as chloroplasts in Figure 1C were retrieved prior to any rarefaction.
To support taxonomic affiliation of ASVs using Mothur, we performed phylogenetic placement of ASVs of interest (ASV_1 and ASV_9 affiliated to the genus *Sulfurimonas* and ASV_2 affiliated to *Thiomicrorhabdus*). Reference sequences from 16S rRNA gene nucleic acid sequences were retrieved from GenBank, IMG/M and LPSN, favoring longer sequences above amplicons. Sequences were aligned using MAFFT (v.7.490) with the L-INS-i algorithm [17, 18] and the reference alignment was trimmed with TrimAl (v.1.4.1) [19], using option for automatic detection of optimal thresholds based on gap scores. The best nucleotide substitution model was chosen using Model Finder [20] implemented in IQ-TREE (v.2.2.0) [21], and the model yielding the lowest Bayesian Information Criterion was chosen. For ASV_1 and ASV_9, the unrooted reference tree was computed with the TMVe+R3 model with free rate with three categories and equal base frequencies. The reference alignment had 1335 bp. Bootstrap values were computed based on 1000 repetitions using the UFBootstrap algorithm [21, 22]. For ASV_2, unrooted reference tree was computed with the GTR+F+I+G4 model with gamma shape alpha=0.264. Proportion of invariant sites: 49%. The reference alignment included 1399 bp. Unrooted maximum likelihood reference trees were computed as the consensus tree after 1000 bootstrap repetitions, using the ultrafast bootstrap method implemented in IQ-TREE [21, 22]. ASV sequences of interest were aligned to the reference alignment using MAFFT with the --addfragments option [23], and were placed on the reference tree using IQ-TREE with the reference tree as constraint. Visualization of the final tree was done after midpoint rooting using iTOL (v5) [24]. Likelihood Weight Ratios of phylogenetic placements were computed using EPA-ng [25] (**Supplementary Table 1**).

Supporting Figures

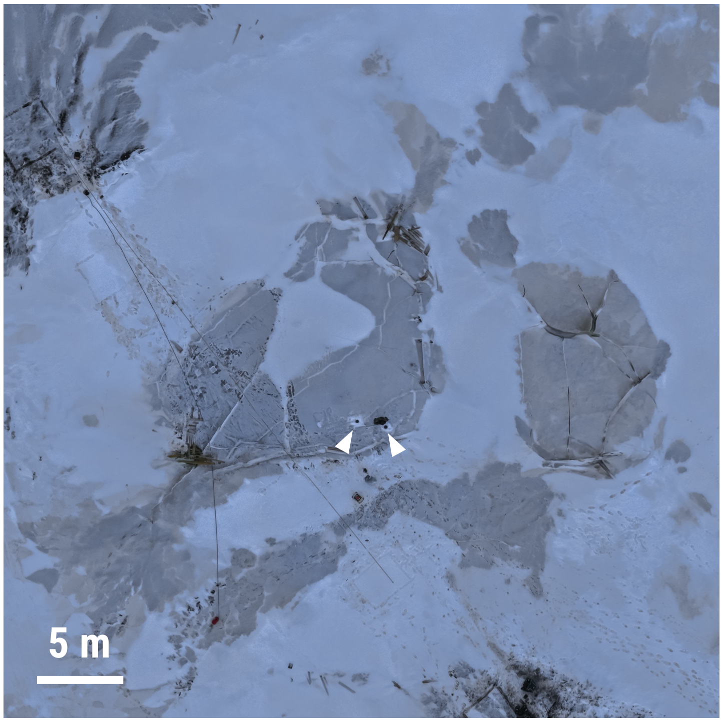

Supplementary Figure 1. Drone image of the Lagoon Pingo during ice core sampling in 2021. The white arrows indicate two boreholes drilled on the main ice blister under which the groundwater seeps.

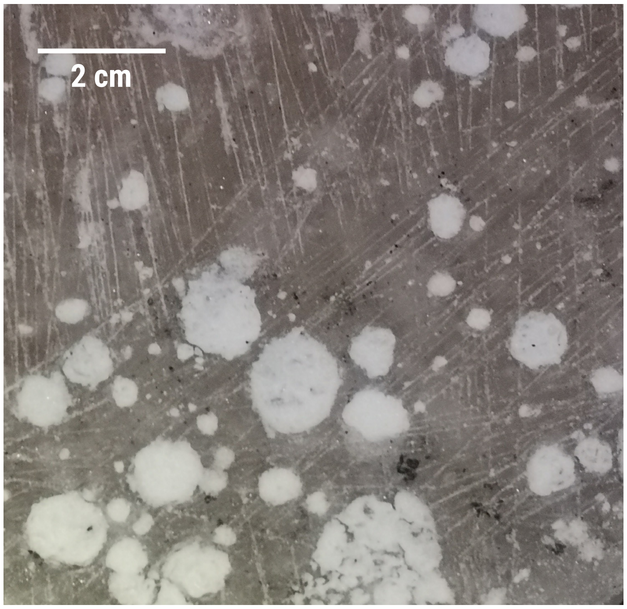

Supplementary Figure 2. Picture of the surface of an ice section originating from an ice core sampled from the ice shell of Lagoon Pingo in February 2024.

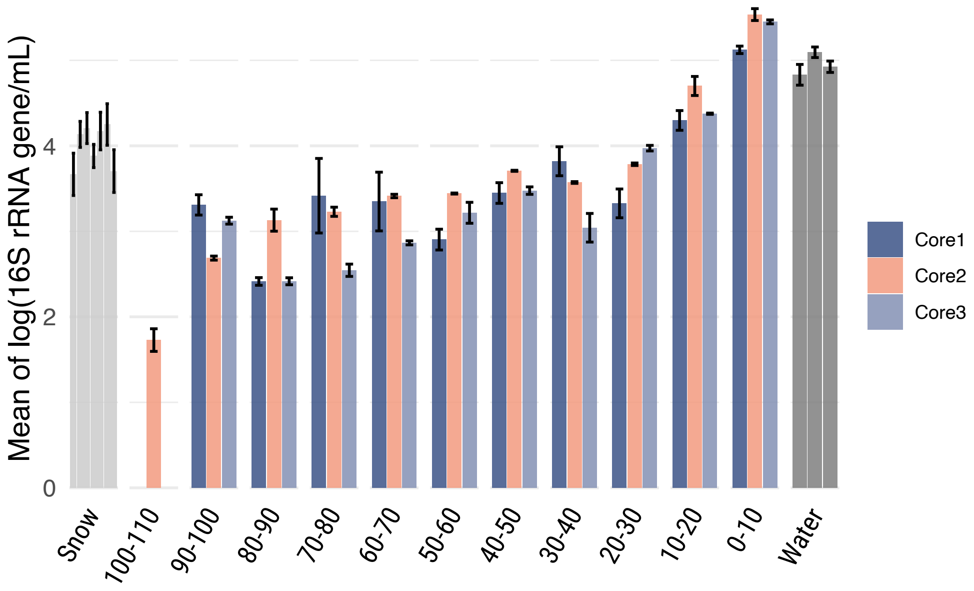

Supplementary Figure 3. Concentration of the 16S rRNA gene per sample, as quantified with quantitative PCR. The values represent the mean of the logarithm of the number of 16S rRNA gene number per mL of melted ice, snow, or water for triplicates. Error bars represent the standard deviation of the same statistic.

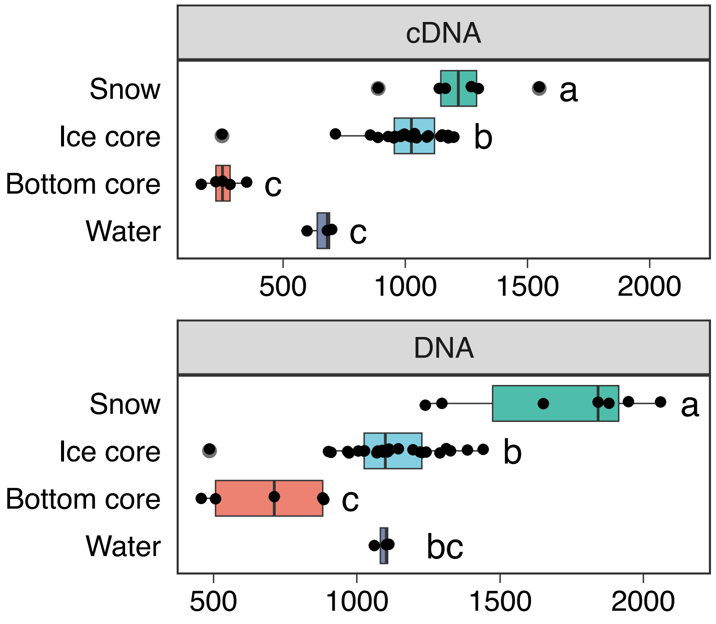

Supplementary Figure 4. Observed ASV numbers in the four environments described in main Figure 1 and in Statistical Analysis of this SI. Letters indicate groups of significance (FDR-corrected *P* value < 0.05) representing Dunn's test output after Kruskal-Wallis significant tests.

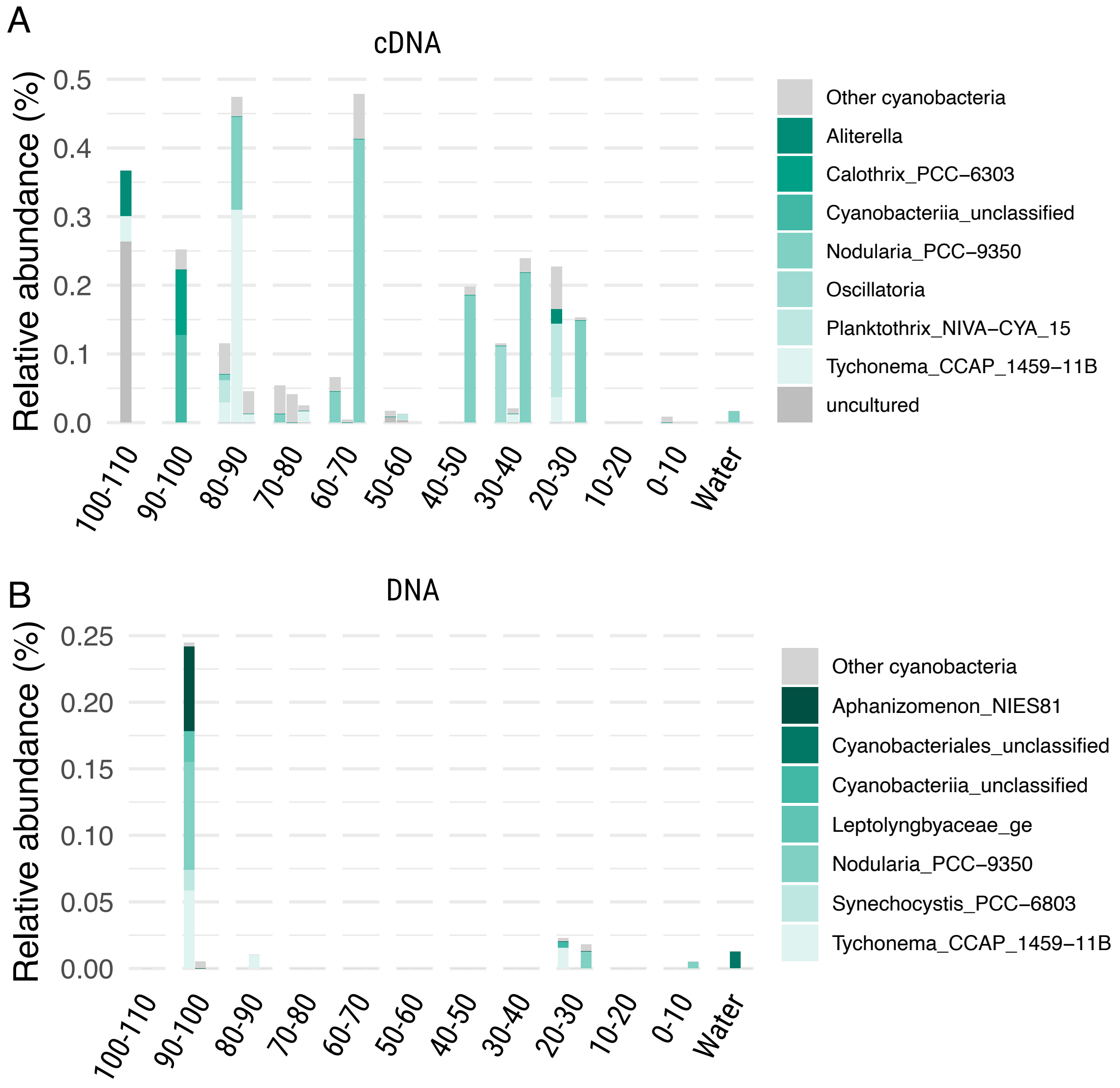

Supplementary Figure 5. Relative abundances of the phylum Cyanobacteria within the ice cores and the water reservoir. The 10 most abundant ASVs among Cyanobacteria are shown at the genus level for the cDNA (**A**) and DNA (**B**) datasets. The other ASVs belonging to the phylum *Cyanobacteria* are shown as “Other cyanobacteria”. The *x* axis indicates the depth of the ice (cm) relative to the water-ice interface (0 cm).

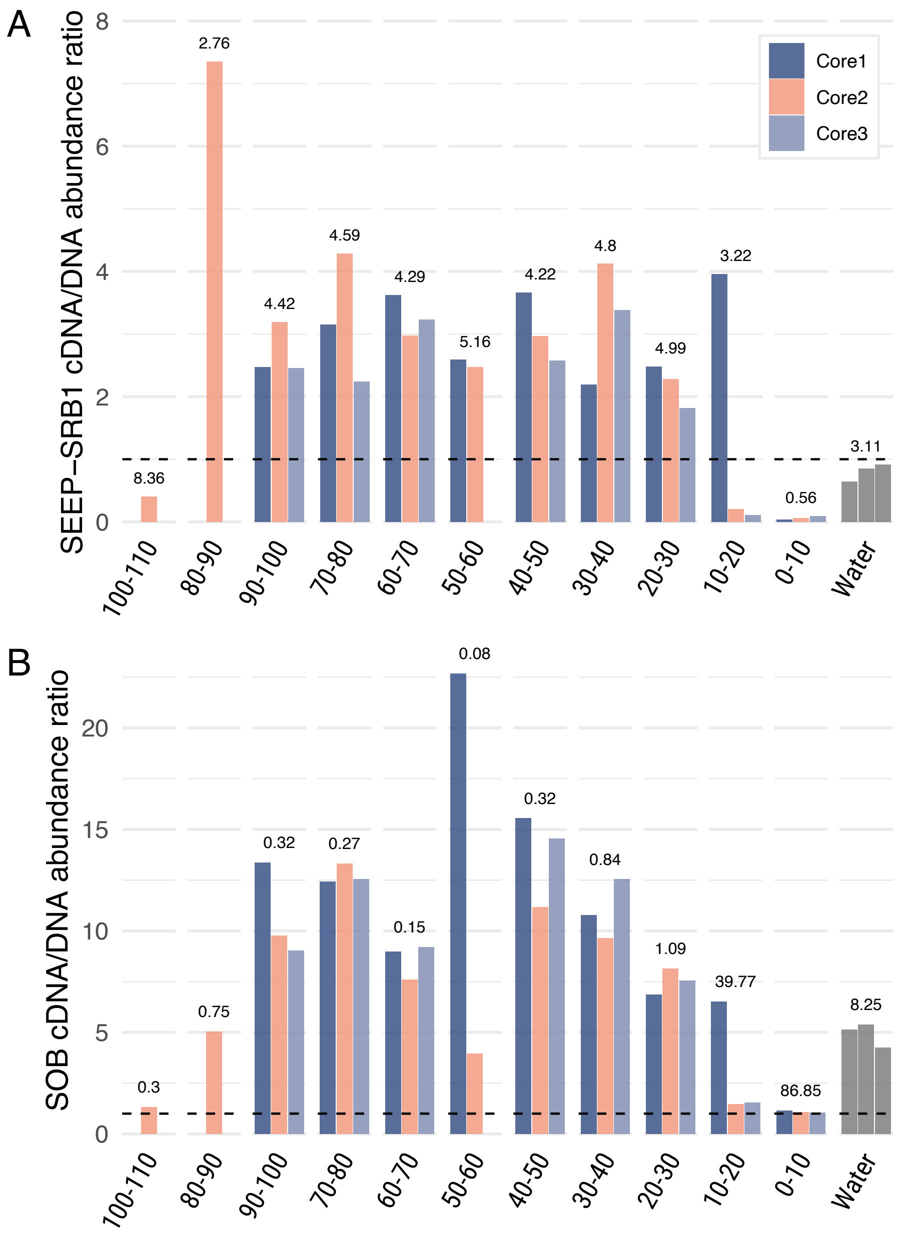

Supplementary Figure 6. (**A**) SEEP-SRB1 cDNA/DNA discrepancy as the ratio of cDNA to DNA relative abundances of ASVs affiliated to SEEP-SRB1 at the genus level. (**B**) SOB cDNA/DNA discrepancy as the ratio of cDNA to DNA relative abundances of ASV_1, ASV_2 and ASV_9 affiliated to the genera *Sulfurimonas* and *Thiomicrorhabdus*. The dashed line represents a ratio of 1 interpreted as no discrepancy. The *x* axis indicates the depth of the ice (cm) relative to the water-ice interface (0 cm). Numbers above each group of bars indicate the mean relative abundance (DNA) within the corresponding depth.

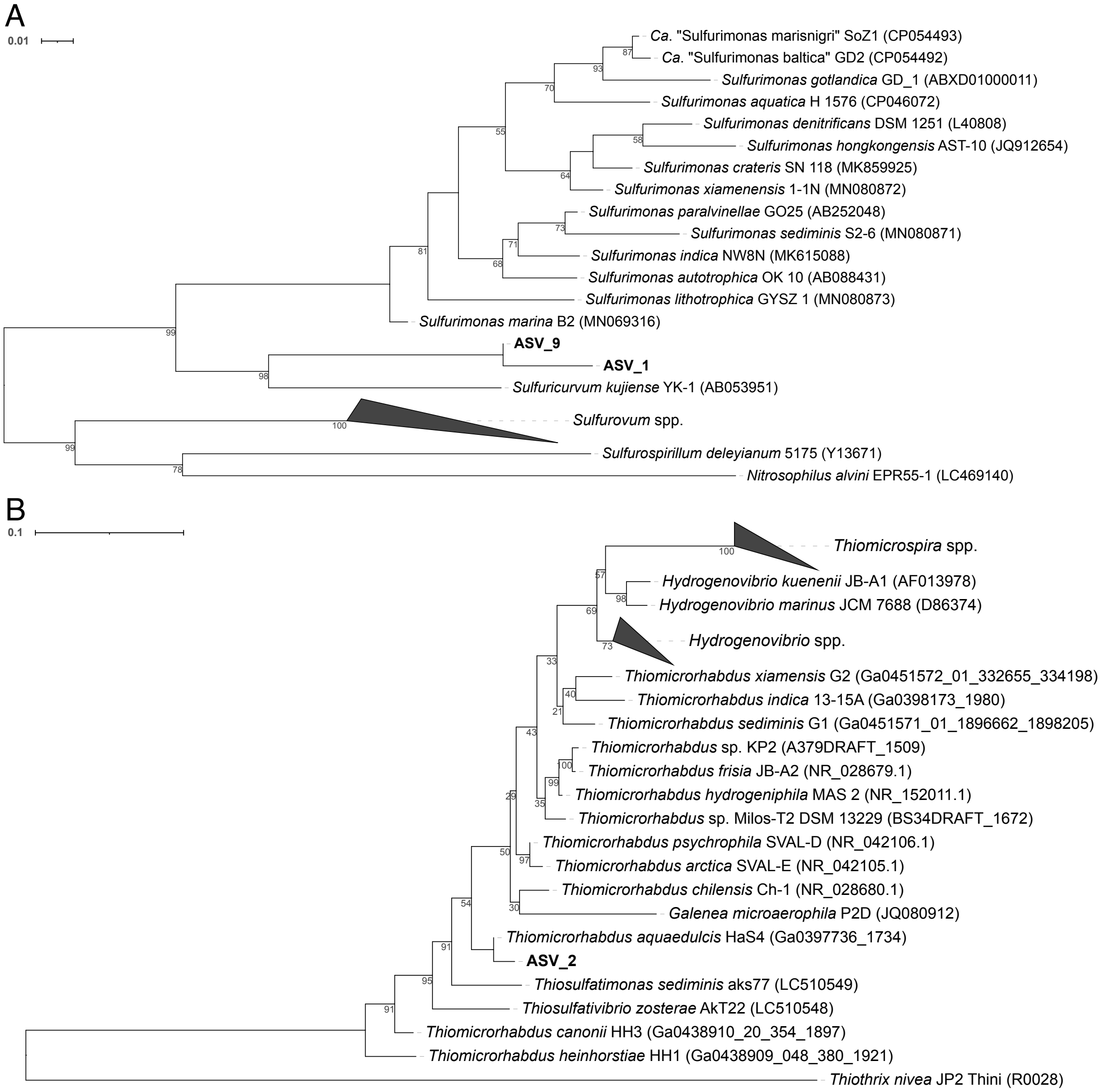

Supplementary Figure 7. Phylogenetic placement of the three ASVs affiliated to the sulfur oxidizing genera *Sulfurimonas* and *Thiomicrorhabdus*. ASVs were placed on a constraint tree topology using IQ-TREE. The tree was built with reference sequences of 16S rRNA gene sequences retrieved from LPSN, IMG/ER and GenBank. Long sequences were preferred over amplicons to construct the constraint tree. The final tree was rooted to midpoint for visualization. (**A**) Phylogenetic placement of ASVs affiliated to the genus *Sulfurimonas* (ASV_1 and ASV_9). The unrooted reference tree was computed with the TMVe+R3 model with free rate with three categories and equal base frequencies. The reference alignment had 1335 bp. Bootstrap values were computed based on 1000 repetitions. Only bootstrap values over 50% are represented. Numbers in parentheses refer to 16S rRNA gene sequence accession number from LPSN. The phylogenetic placement of *Sulfurimonas* ASVs was not conclusive. Likelihood Weight Ratios computed using EPA-ng [25] are available in **Supplementary Table 1**. (**B**) Phylogenetic placement for the ASV affiliated to the genus *Thiomicrorhabdus* (ASV_2). The unrooted reference tree was computed with the GTR+F+I+G4 model with gamma shape alpha = 0.264. Proportion of invariant sites: 49%. The reference alignment included 1399 bp. Numbers in parentheses refer to 16S rRNA gene sequence accession number from GenBank (beginning with NR_), LPSN (one sequence numbers), or IMG/ER (long/containing underscores). Scale bars: nucleotide substitutions per site.

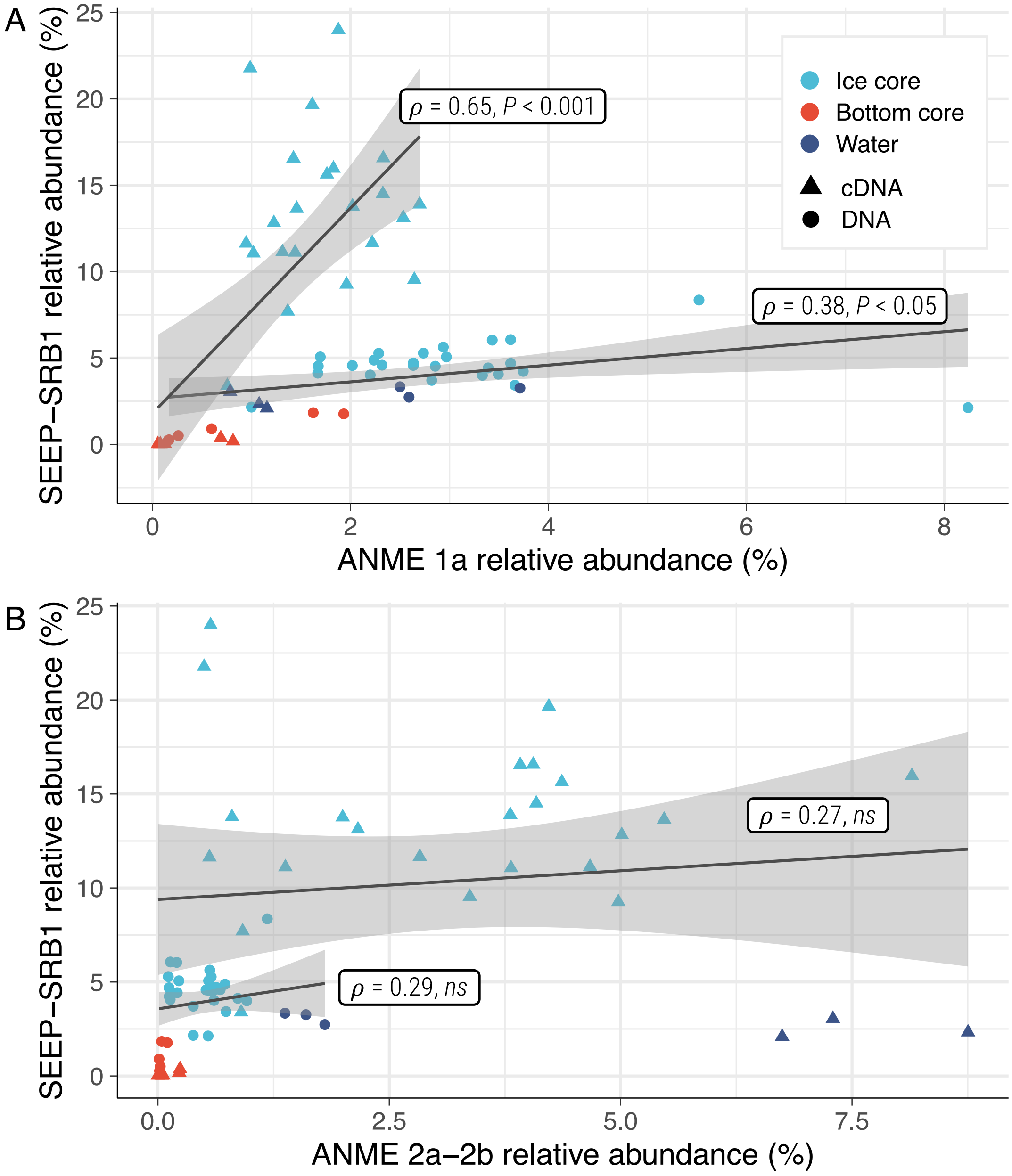

Supplementary Figure 8. Spearman correlation plots between relative abundances of SEEP-SRB1 and (**A**) ANME 1a and (**B**) ANME 2a-2b. Colors indicate the sample's environment and shapes the dataset (cDNA or DNA). For visualization purposes the black lines depict linear models of the regressions with a 95% confidence interval (grey area). Correlations were tested using Spearman's rank test, after verifying that the data are not normally distributed. Labels give Spearman's rho and the associated test *P* value. *ns*: not significant (*P* value > 0.1).

Supporting Tables

Supplementary Table 1. EPA-ng results for ASVs affiliated to the genera *Sulfurimonas* (ASV_1 and ASV_9) and *Thiomicrorhabdus* (ASV_2).

ASV	Likelihood	Likelihood Weight Ratio	Distal length	Pendant length	Node
ASV_2	-1653,4	0,994	0,000	0,016	19
ASV_1	-1992,1	0,019	0,073	0,110	10
ASV_1	-1989,3	0,326	0,032	0,099	35
ASV_1	-1989,3	0,326	0,084	0,099	36
ASV_1	-1989,3	0,327	0,000	0,099	34
ASV_9	-1981,1	0,085	0,089	0,087	10
ASV_9	-1979,9	0,269	0,032	0,084	35
ASV_9	-1979,1	0,597	0,036	0,105	34
ASV_9	-1981,6	0,047	0,007	0,086	33

Supporting Information References

1. Hodson AJ, Nowak A, Redeker KR, Holmlund ES, Christiansen HH, Turchyn AV. Seasonal dynamics of methane and carbon dioxide evasion from an open system pingo: Lagoon Pingo, Svalbard. *Front Earth Sci* 2019; **7**: 30.

2. Cruaud P, Vigneron A, Fradette M-S, Charette SJ, Rodriguez MJ, Dorea CC, et al. Open the SterivexTM casing: an easy and effective way to improve DNA extraction yields. *Limnol Oceanogr Methods* 2017; **15**: 1015–1020.

3. Angel R, Claus P, Conrad R. Methanogenic archaea are globally ubiquitous in aerated soils and become active under wet anoxic conditions. *ISME J* 2012; **6**: 847–862.

4. Parada AE, Needham DM, Fuhrman JA. Every base matters: assessing small subunit rRNA primers for marine microbiomes with mock communities, time series and global field samples. *Environ Microbiol* 2016; **18**: 1403–1414.

5. Callahan BJ, McMurdie PJ, Rosen MJ, Han AW, Johnson AJA, Holmes SP. DADA2: High-resolution sample inference from Illumina amplicon data. *Nat Methods* 2016; **13**: 581–583.

6. Schloss PD, Westcott SL, Ryabin T, Hall JR, Hartmann M, Hollister EB, et al. Introducing mothur: open-source, platform-independent, community-supported software for describing and comparing microbial communities. *Appl Environ Microbiol* 2009; **75**: 7537–7541.

7. Quast C, Pruesse E, Yilmaz P, Gerken J, Schweer T, Yarza P, et al. The SILVA ribosomal RNA gene database project: improved data processing and web-based tools. *Nucleic Acids Res* 2013; **41**: D590–596.

8. Suzuki MT, Taylor LT, DeLong EF. Quantitative analysis of small-subunit rRNA genes in mixed microbial populations via 5′-nuclease assays. *Appl Environ Microbiol* 2000; **66**: 4605–4614.

9. Spangenberg I, Overduin PP, Damm E, Bussmann I, Meyer H, Liebner S, et al. Methane pathways in winter ice of a thermokarst lake–lagoon–coastal water transect in north Siberia. *Cryosphere* 2021; **15**: 1607–1625.

10. R Core Team. R: A language and environment for statistical computing. 2021. R Foundation for Statistical Computing, Vienna, Austria.

11. McMurdie PJ, Holmes S. Waste not, want not: why rarefying microbiome data is inadmissible. *PLoS Comput Biol* 2014; **10**: e1003531.

12. Oksanen J, Blanchet FG, Friendly M, Kindt R, Legendre P, McGlinn D, et al. *vegan*: community ecology package. R package version 2.5-7. 2020.

13. Legendre P, Gallagher ED. Ecologically meaningful transformations for ordination of species data. *Oecologia* 2001; **129**: 271–280.

14. Anderson MJ. Permutational Multivariate Analysis of Variance (PERMANOVA). *Wiley StatsRef: Statistics Reference Online*. 2017. American Cancer Society, pp 1–15.

15. Martinez Arbizu P. *pairwiseAdonis*: Pairwise multilevel comparison using adonis. R package version 0.4. 2020.

16. Wickham H. *ggplot2*: elegant graphics for data analysis. Springer-Verlag New York. 2016.

17. Katoh K, Kuma K, Toh H, Miyata T. MAFFT version 5: improvement in accuracy of multiple sequence alignment. *Nucleic Acids Res* 2005; **33**: 511–518.

18. Katoh K, Standley DM. MAFFT Multiple sequence alignment software version 7: improvements in performance and usability. *Mol Biol Evol* 2013; **30**: 772–780.

19. Capella-Gutiérrez S, Silla-Martínez JM, Gabaldón T. trimAl: a tool for automated alignment trimming in large-scale phylogenetic analyses. *Bioinformatics* 2009; **25**: 1972–1973.

20. Kalyaanamoorthy S, Minh BQ, Wong TKF, von Haeseler A, Jermiin LS. ModelFinder: fast model selection for accurate phylogenetic estimates. *Nat Methods* 2017; **14**: 587–589.

21. Nguyen L-T, Schmidt HA, von Haeseler A, Minh BQ. IQ-TREE: A fast and effective stochastic algorithm for estimating maximum-likelihood phylogenies. *Mol Biol Evol* 2015; **32**: 268–274.

22. Hoang DT, Chernomor O, von Haeseler A, Minh BQ, Vinh LS. UFBoot2: Improving the ultrafast bootstrap approximation. *Mol Biol Evol* 2018; **35**: 518–522.

23. Katoh K, Frith MC. Adding unaligned sequences into an existing alignment using MAFFT and LAST. *Bioinformatics* 2012; **28**: 3144–3146.

24. Letunic I, Bork P. Interactive Tree Of Life (iTOL) v5: an online tool for phylogenetic tree display and annotation. *Nucleic Acids Res* 2021; **49**: W293–W296.

25. Barbera P, Kozlov AM, Czech L, Morel B, Darriba D, Flouri T, et al. EPA-ng: massively parallel evolutionary placement of genetic sequences. *Syst Biology* 2019; **68**: 365–369.
